# Whole blood RNA sequencing reveals a unique transcriptomic profile in patients with ARDS following hematopoietic stem cell transplantation

**DOI:** 10.1186/s12931-019-0981-6

**Published:** 2019-01-21

**Authors:** Joshua A. Englert, Michael H. Cho, Andrew E. Lamb, Maya Shumyatcher, Diana Barragan-Bradford, Maria C. Basil, Angelica Higuera, Colleen Isabelle, Mayra Pinilla Vera, Paul B. Dieffenbach, Laura E. Fredenburgh, Joyce B. Kang, Ami S. Bhatt, Joseph H. Antin, Vincent T. Ho, Robert J. Soiffer, Judie A. Howrylak, Blanca E. Himes, Rebecca M. Baron

**Affiliations:** 1 Division of Pulmonary, Critical Care, and Sleep Medicine, The Ohio State Wexner Medical Center, 201 Davis Heart and Lung Research Institute, 473 West 12th Avenue, Columbus, OH 43210 USA; 2 Channing Division of Network Medicine, Brigham and Women’s Hospital, Harvard Medical School, 181 Longwood Avenue, Boston, MA 02115 USA; 3 Division of Pulmonary and Critical Care Medicine, Brigham and Women’s Hospital, Harvard Medical School, 75 Francis Street, Boston, MA 02115 USA; 4 Department of Biostatistics, Epidemiology and Informatics, University of Pennsylvania, 402 Blockley Hall, 423 Guardian Drive, Philadelphia, PA 19104 USA; 5 Departments of Medicine and Genetics, Stanford University, CCSR1155b, 269 Campus Drive, Palo Alto, CA 93405 USA; 6 Division of Hematologic Malignancies, Dana-Farber Cancer Institute, Harvard Medical School, 450 Brookline Avenue, Boston, MA 02215 USA; 7 Division of Pulmonary and Critical Care Medicine, Penn State Milton S. Hershey Medical Center, 500 University Drive, Hershey, PA 17033 USA

**Keywords:** Acute respiratory distress syndrome (ARDS), RNA sequencing, RNA-Seq, Transcriptome profiling, Hematopoietic stem cell transplantation, Respiratory failure, Bone marrow transplant

## Abstract

**Background:**

The acute respiratory distress syndrome (ARDS) is characterized by the acute onset of hypoxemia and bilateral lung infiltrates in response to an inciting event, and is associated with high morbidity and mortality. Patients undergoing allogeneic hematopoietic stem cell transplantation (HSCT) are at increased risk for ARDS. We hypothesized that HSCT patients with ARDS would have a unique transcriptomic profile identifiable in peripheral blood compared to those that did not undergo HSCT.

**Methods:**

We isolated RNA from banked peripheral blood samples from a biorepository of critically ill ICU patients. RNA-Seq was performed on 11 patients with ARDS (5 that had undergone HSCT and 6 that had not) and 12 patients with sepsis without ARDS (5 that that had undergone HCST and 7 that had not).

**Results:**

We identified 687 differentially expressed genes between ARDS and ARDS-HSCT (adjusted *p*-value < 0.01), including *IFI44L*, *OAS3, LY6E,* and *SPATS2L* that had increased expression in ARDS vs. ARDS-HSCT; these genes were not differentially expressed in sepsis vs sepsis-HSCT. Gene ontology enrichment analysis revealed that many differentially expressed genes were related to *response to type I interferon*.

**Conclusions:**

Our findings reveal significant differences in whole blood transcriptomic profiles of patients with non-HSCT ARDS compared to ARDS-HSCT patients and point toward different immune responses underlying ARDS and ARDS-HSCT that contribute to lung injury.

**Electronic supplementary material:**

The online version of this article (10.1186/s12931-019-0981-6) contains supplementary material, which is available to authorized users.

## Background

ARDS is a syndrome with high morbidity and mortality characterized by the acute onset of bilateral lung infiltrates and hypoxemia that frequently results in acute respiratory failure [1]. A recent study demonstrated that the worldwide burden of ARDS is greater than previously appreciated and accounts for up to 10% of intensive care unit (ICU) admissions [2]. Despite decades of research into the pathophysiology underlying ARDS, supportive care with mechanical ventilation remains the mainstay of therapy, and there are no effective targeted pharmacologic treatments for these patients. One barrier to developing effective therapies is that ARDS is a heterogeneous syndrome that encompasses a wide variety of patients with lung injury from many different causes. We and other groups have postulated that the pathophysiology of lung injury in ARDS subgroups may differ substantially which, in turn, may lead to variable responses in ARDS clinical trials [3–6]. Recently, several studies have used clinical data and previously identified biomarkers to define several ARDS subphenotypes with distinct clinical outcomes, inflammatory profiles, and response to ARDS therapies [3, 7, 8] underscoring the need for a better understanding of these different populations.

One important subgroup of patients at increased risk for ARDS that experience particularly high mortality are those with hematologic malignancies undergoing hematopoietic stem cell transplantation. While allogeneic hematopoietic stem cell transplantation (allo-HSCT) is a life-saving treatment for patients with hematologic malignancies [9], a variety of pulmonary complications, both infectious and non-infectious, can complicate the post-transplant course [10, 11]. One report found that ARDS occurs in over 15% of patients undergoing allo-HSCT with ICU mortality rates of nearly 50% [12]. It remains unclear whether the pathobiology of ARDS following HSCT is similar to ARDS in other patient populations, even though ARDS post HSCT is often clinically indistinguishable from ARDS in non-HSCT patients. A better understanding of the biology of ARDS following allo-HSCT may lead to novel strategies for prevention and treatment in this population and, in addition, might identify broad treatment approaches for ARDS.

Previous transcriptomic studies have used expression microarrays to search for ARDS gene-expression signatures. Using whole blood mRNA, Howrylak et al. identified a signature of 8 genes in ICU patients with sepsis that was associated with the development of ARDS [13]. A microarray study of septic patients found increased expression of neutrophil related genes early in ARDS [14], and our group demonstrated the critical role of IL18, a prominent inflammasome-related cytokine, in the pathogenesis of sepsis-induced ARDS [15]. In a study by Juss et al., peripheral blood neutrophils from ARDS patients were compared to those of healthy volunteers, and patients with ARDS had significant increases in immune response pathways [16]. Published ARDS transcriptomic studies have not characterized different subgroups of patients with ARDS, an approach that may reveal distinct gene expression changes that underlie similar clinical symptoms. Here, we used RNA-Seq, a powerful and unbiased approach to characterize transcriptomes, to compare gene expression in the blood of patients with ARDS following allo-HSCT compared to patients with ARDS that had not undergone allo-HSCT.

## Methods

### Study design and sample collection

**Fig. 1:**
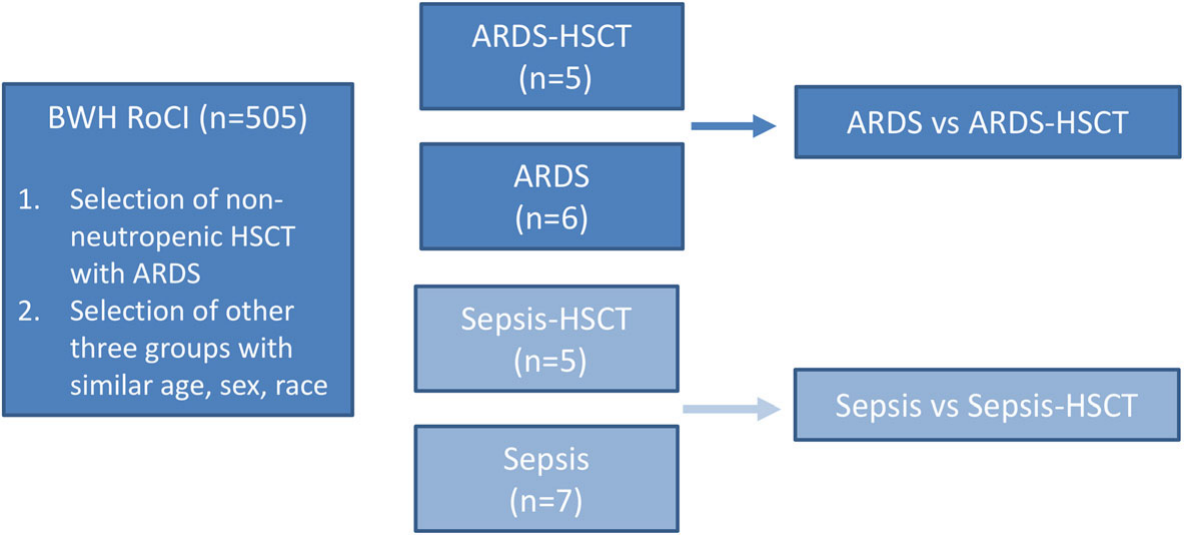
Schematic diagram of the experimental design. The primary analysis compared transcriptomic profiles of patients with ARDS compared to ARDS-HSCT (dark blue). In order to identify differences that were due to HSCT status, transcripts that were differentially regulated in sepsis patients compared to sepsis-HSCT (light blue) were excluded from the analysis

Patients with ARDS following HSCT who contributed samples to the IRB-approved Brigham and Women’s Registry of Critical Illness (RoCI) from 2008 to 2013 were identified [6, 17, 18]. All patients were admitted to the medical intensive care unit at Brigham and Women’s Hospital at the time of enrollment in the RoCI. Subjects with ARDS that did not undergo HSCT from the same 5 year period were then identified as a comparator group. To reduce sample heterogeneity, we selected subjects of similar age, time in the ICU, male sex, and white race. ARDS was defined by the Berlin [1] definition for cases after 2012 and the American-European Consensus (AECC) definition [19] for cases prior to 2012. While our primary interest was identifying differences in gene expression in ARDS subjects that had undergone HSCT compared those without HSCT, we also sequenced 12 subjects with sepsis (HSCT and non-HSCT) without ARDS in order to identify differences between the ARDS groups that were not due to HSCT status. Sepsis was defined by the presence of the systemic inflammatory response syndrome (SIRS) with suspected or confirmed infection [20]. Whole blood samples from the beginning of the ICU course (within the first 5 days of ICU admission) were used for gene expression profiling. Our overall study design is shown in Fig. 1.

### RNA-Seq library construction and sequencing

RNA was extracted and globin-cleared using the Ambion GLOBINclear kit (Thermo Fisher, Waltham, MA). RNA-Seq libraries were prepared with 0.1–1 μg total RNA using Illumina TruSeq RNA-Seq v2 kit (Illumina, San Diego, CA) according to manufacturer protocol. Ambion External RNA Controls Consortium (ERCC) RNA Spike-In Control Mix 1 (Thermo Fisher, Waltham, MA) was added to the samples. Quality control of libraries involved picogreen and size analysis on an Agilent Bioanalyzer or Tapestation 2200 (Agilent, Santa Clara, CA) and qPCR quantitation against a standard curve. Samples with an initial low RIN (i.e. < 6) were re-extracted; any samples with a persistent RIN < 6 were run in duplicate. Samples were randomized to one of 4 pools of 5–6 subjects, and each pool was sequenced in two different lanes. Sequencing of 75 base pair, paired-end reads was performed with an Illumina HiSeq 2500 instrument at Partners Personalized Medicine (Boston, MA) under rapid mode using PhiX spike-in and processed using Real-Time Analysis v1.18.64, Control Software v2.2.58 (Illumina, San Diego, CA).

### RNA-Seq data analysis

Preliminary processing of raw reads was performed using Casava 1.8 (Illumina, San Diego, CA). Reads were aligned to the human genome build 38 with STAR v2.4.0. [21] Taffeta scripts (https://github.com/blancahimes/taffeta) were used to assess quality of aligned reads, which included quantifying number of mapped reads, junction spanning reads, assessing 5′ and 3′ bias, and insert size distribution. For each sample, ERCC Spike-in dose response curves (i.e. plots of ERCC transcript FPKM vs. ERCC transcript molecules) were created following the manufacturer’s protocol [22]. Because samples with low RIN (< 6) had characteristics similar to those of higher quality and duplicate samples were highly correlated, we combined data for duplicate samples and included them in further analyses. Raw read plots were created by displaying bigwig files for each sample in the UCSC Genome Browser. The RNA-Seq data is available at the Gene Expression Omnibus Web site (http://www.ncbi.nlm.nih.gov/geo/) under accession GSE84439. Gene counts were obtained with HTSeq v0.6.1. Differential expression analysis was performed using DESeq2 v1.12.3 [23], after excluding reads mapped to hemoglobin genes and while adjusting for RIN scores as a covariate [24]. The NIH Database for Annotation, Visualization and Integrated Discovery (DAVID) was used to perform gene ontological category enrichment analysis using *Homo sapiens* as background, and default options and annotation categories [25].

### qPCR validation of differentially expressed genes

RNA (200 ng per subject) was used to prepare cDNA utilizing the Applied Biosciences High Capacity cDNA Reverse Transcriptase Kit in 20 μl reactions (Thermo Fisher, Waltham, MA). The cDNA was then used for qPCR analysis with the following Taqman/Applied Biosciences (Thermo Fisher, Waltham, MA) primers (all human and all primers chosen to span an exon junction): *IFI44L* (Hs00915292_m1), *OAS2* (Hs00942643_m1), *OAS3* (Hs00196324_m1), *SPATS2L* (Hs91916364_m1). Briefly, qPCR was performed with a 1:5 dilution of cDNA and run for 40 cycles. All samples were run in triplicate, and GAPDH was used as a housekeeping control. All samples had SD < 0.5 between triplicates. Controls without cDNA templates, primers, and reverse transcriptase were included and were all appropriately negative. Delta CT values between genes of interest and GAPDH were analyzed for statistical significance using a t-test [26].

### Analysis of unmapped reads

Fastq files containing paired-end reads were merged for each sample (i.e., forward and reverse sequence files were combined). Reads were trimmed with Trim Galore using a minimum Phred quality score of 20 and minimum final read length of 60 bp [27]. Trimmed reads were de-duplicated to remove PCR artifacts and sequences containing at least one “N” was performed with Super-Deduper [28]. Reads of potential human origin were removed by aligning resultant fastq files to GRCh37 with STAR using the recommended default parameters [21]. The unmapped (non-human) reads were taxonomically classified based on mapping all *k*-mers (k = 31) within each sequence to the lowest common ancestor in a database of > 83,000 bacterial, viral, fungal, and protozoan genomes in the OneCodex reference database (database version as of February 26, 2018). Default OneCodex alignment parameters were used [29].

## Results

### Subject characteristics

Characteristics of all subjects are shown in Table 1. To reduce sources of gene expression heterogeneity not related to disease status, selected subjects were age-matched, non-Hispanic white males with similar WBC, APACHE scores, and severity of hypoxemia (PaO2/FiO2 ratios) (Wilcoxon *P* > 0.1). For the HSCT-ARDS group, all patients had undergone HSCT for the treatment of various forms of acute leukemia and median time from transplant to development of ARDS was 117 days. All patients with ARDS received supportive care with low tidal volume ventilation and average tidal volumes did not differ between the ARDS and ARDS-HSCT at the time that blood samples were collected (Table 1). None of the ARDS subjects were treated with neuromuscular blockade or prone positioning at the time the blood sample used for RNA sequencing was collected. All HSCT-ARDS subjects were treated with corticosteroids (median dose 80 mg methylprednisolone daily), as were half of ARDS subjects (median dose 16 mg methylprednisolone daily). Similarly, in the non-ARDS control groups with sepsis, all of the sepsis-HSCT and half of the sepsis subjects were treated with corticosteroids. Etanercept was given to 4 of the 5 ARDS-HSCT patients as treatment for idiopathic pneumonia syndrome. Two ARDS subjects were confirmed to have influenza infection (Table 1). Patients with sepsis were managed with source control, early antibiotic administration, and volume resuscitation in accordance with sepsis guidelines at the time [30, 31].

**Table 1:** Baseline characteristics of the subjects

Characteristics	ARDS	ARDS-HSCT	Sepsis	Sepsis-HSCT	P (all groups)	P (ARDS vs ARDS-HSCT)
N	6	5	7	5		
Age	46 (37–60)	39 (25–52)	50 (26–59)	55 (61–42)	0.27	0.41
WBC	6.9 (3.1–16.8)	11 (2.8–19.1)	9 (4–42.1)	8.6 (6.5–29.9)	0.88	0.86
% Neutrophils	87 (71–98)	95 (88–98)	86 (63–95)	80 (65–96)	0.14	0.10
% Lymphocytes	6 (1–18)	2 (0–10)	10 (1–21)	10 (1–21)	0.26	0.12
% Monocytes	4 (1–15)	2 (0–4)	4 (1–11)	9 (0–11)	0.39	0.46
Sepsis	33.3%	40.0%	100%	100%		1
Vasopressors	50%	60%	29%	40%	0.8	1
Days since HSCT		117 (50–490)		148 (50–1322)		
APACHE II	27.5 (21–47)	30.0 (24–38)	18 (14–40)	19 (14–32)	0.10	0.58
ICU Mortality	67%	100%	14%	0%	0.001	0.45
Mechanical Ventilation	100%	100%	29%	40%	0.04	1
P/F ratio	70.0 (41–205.0)	81.3 (66.0–109.2)				0.83
Tidal volume (mL/kg)	5.6 (5.2–7.6)	6.5 (4.5–16.5)				0.46
Corticosteroids	50%	100.0%	57%	100%	0.04	0.18
Etanercept	0.0%	80.0%	0%	0%		0.02
Other Immunosuppression	50.0%	80%	0%	60%	0.02	0.5
ARDS etiology						
Pneumonia, influenza	33%	0%				
Pancreatitis	17%	0%				
Idiopathic pneumonia syndrome	0%	100%				
Idiopathic/CVD	50%	0%				
Infection						
Type						
Bacterial	0%	0%	100%	80%		
Viral	33%	0%	0%	20%		
Source						
Blood	0%	0%	43%	60%		
Lung	33%	0%	14%	20%		
GI	0%	0%	57%	0%		

Idiopathic/collagen-vascular disease (CVD) diagnoses include acute interstitial pneumonia, cryptogenic organizing pneumonia, and collagen-vascular disease associated interstitial lung disease. Values given as median (range)

### Immune response and interferon-stimulated genes are upregulated in ARDS compared to ARDS-HSCT

**Fig. 2:**
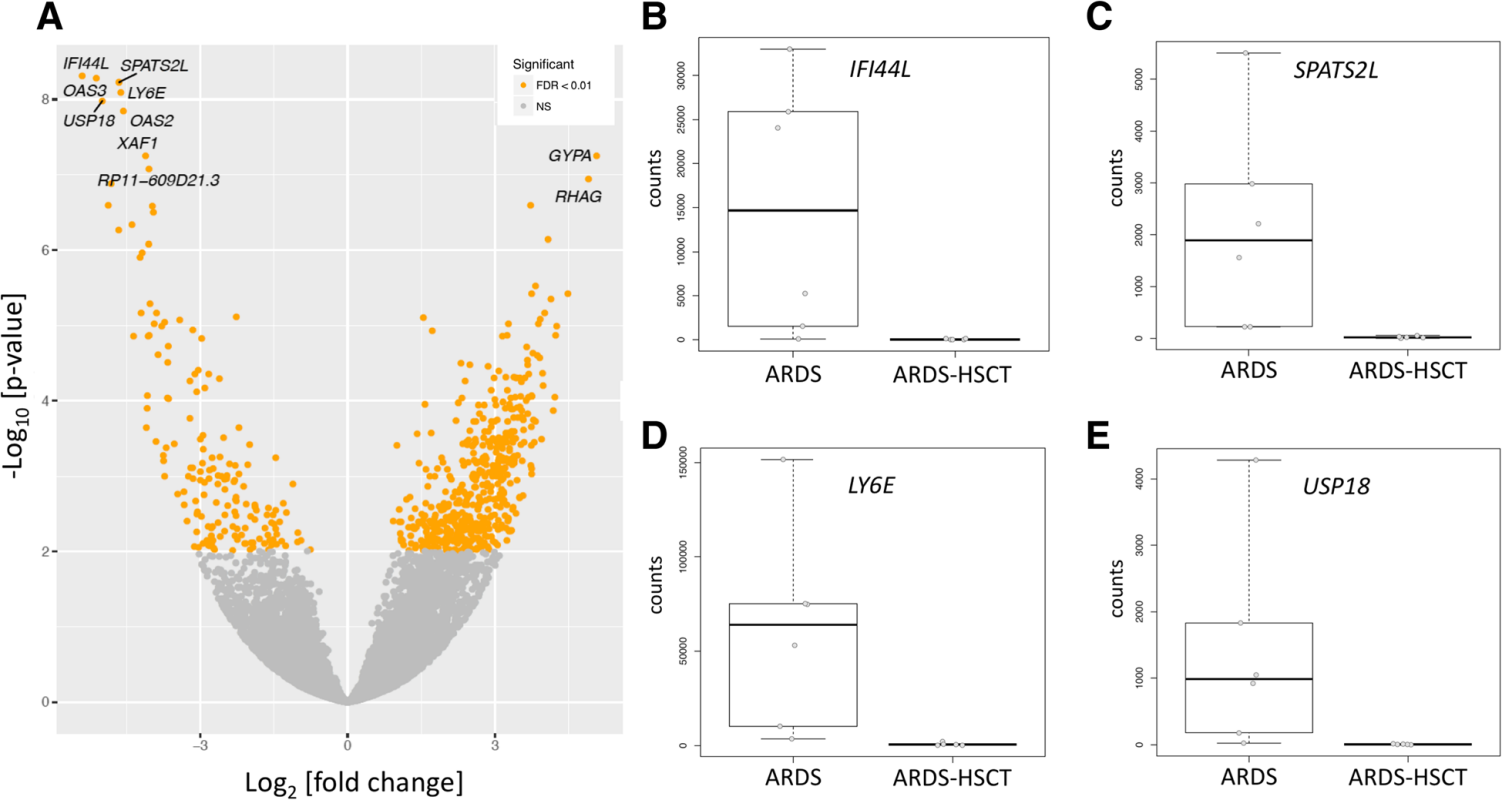
Results of differential expression analysis. **a**) Volcano plot of overall gene-based differential expression results of subjects with ARDS vs ARDS-SCT. The x-axis corresponds to the log (base 2) of the fold change difference between groups and the y-axis corresponds to the negative log (base 10) of the *p*-values. There were 687 differentially expressed genes (yellow dots) when using an adjusted *p*-value < 0.01. **b**-**e**) Boxplots of several of the most differentially expressed transcripts

We identified 687 differentially expressed genes between ARDS and ARDS-HSCT (false-discovery rate (FDR) corrected *p*-value < 0.01). In ARDS-HSCT vs. ARDS, we found 687 differentially expressed genes (520 were upregulated, 167 were downregulated, Fig. 2). To identify which differences in gene expression reflected unique features of ARDS-HSCT, rather than the history of HSCT itself, we filtered out genes that were also nominally differentially expressed (unadjusted p-value < 0.1) in a consistent direction in patients with sepsis-HSCT vs. sepsis. Of the 687 differentially expressed genes, 483 were not present in the sepsis-HSCT vs. sepsis comparison, suggesting that the majority of expression changes reflected processes unique to ARDS-HSCT. Top differentially expressed genes upregulated in ARDS vs. ARDS-HSCT included immune response and interferon signaling genes (e.g. *IFI44L*, *OAS3*, *LY6E*, *OAS2, USP18*) as shown in Table 2. Gene ontological category enrichment analysis with all 687 differentially expressed genes found that *immune response-* and *interferon signaling-*related pathways were statistically overrepresented (Table 3 and Additional file 1: Tables S1 to Table S3). To confirm top RNA-Seq results, we selected several of the most differentially expressed genes and performed qRT-PCR using an aliquot of whole blood RNA from the subjects that were sequenced. The qRT-PCR results were consistent with the RNA-Seq results and showed significant differences in expression for the transcripts that were tested (Additional file 2: Figure S1).

**Table 2:** Top 10 most differentially expressed transcripts from ARDS vs ARDS-HSCT

Gene	feature_name	log2FoldChange	Adjusted P
*IFI44L*	ENSG00000137959	−5.4	5.0E-09
*OAS3*	ENSG00000111331	−5.1	5.3E-09
*SPATS2L*	ENSG00000196141	−4.6	6.0E-09
*LY6E*	ENSG00000160932	−4.6	8.1E-09
*USP18*	ENSG00000184979	−5.0	1.1E-08
*OAS2*	ENSG00000111335	−4.6	1.4E-08
*XAF1*	ENSG00000132530	−4.1	5.7E-08
*GYPA*	ENSG00000170180	5.1	5.7E-08
*RP11-609D21.3*	ENSG00000279296	−4.0	8.4E-08
*RHAG*	ENSG00000112077	4.9	1.2E-07

**Table 3:** Top results from DAVID Functional Annotation Clustering

Cluster	Enrichment score	Representative category	Count	Adjusted *P*-value
1	11.05	Antiviral defense	30	3.9 × 10–19
2	5.51	Response to virus	17	1.3 × 10–5
3	3.84	Heme biosynthesis	7	2.8 × 10–5
4	3.29	Hereditary hemolytic anemia	9	4.2 × 1–05
5	3	Herpes simplex infection	16	1.8 × 10–2

### Differences in transcriptomic profiles are not due to influenza infection in ARDS subjects

To determine whether increased expression of immune response pathway genes in ARDS was due to viral infection, we repeated the differential expression analysis after excluding two patients with confirmed influenza infection (remaining ARDS subjects did not have any evidence of active viral infection while in the ICU). Results of this sub-analysis were consistent with those of the full set of subjects: of the top 100 genes differentially expressed in ARDS, 99 out of 100 were also differentially expressed after removal these two patients, and top results of the ontological category enrichment analysis were unchanged (Additional file 1: Table S2). Thus, it appears that increased expression of interferon-related genes in ARDS was not due to influenza infection.

### Analysis of non-human transcripts did not show evidence of undiagnosed infection in patients with ARDS after HSCT

Some experts have speculated that lung injury following stem cell transplant may be due to infection with pathogens that are not identified using microbiologic techniques routinely used in clinical laboratories [32]. For example, next-generation sequencing was used to discover a novel bacterium as a candidate pathogen in cord colitis syndrome, a complication of allogeneic cord blood HSCT [33]. To investigate the possibility that undiagnosed infection accounts for differences observed in ARDS-HSCT, we used RNA-Seq reads that did not map to the human genome to perform genus-level taxonomic classification. After removing possible duplicate reads, we analyzed the remaining 8–19% of unmapped reads. Based on the genera present and relative similarity of microbial profiles across all 23 subjects, there were no significant differences between samples both within and among categories (Additional file 2: Figure S2). Additional file 1: Table S3 shows the number of reads per sample assignable to a virus. We detected the presence of virus in four subjects (with reads mapping to EBV and torquetenovirus). Only one subject had > 10 reads assignable to a virus (with 97 reads aligning to EBV). The lack of recurrent abundant pathogens in the ARDS HSCT population is consistent with clinical data and suggested that these patients did not have undiagnosed bacterial or viral bloodstream infections as a proximate cause of their ARDS.

## Discussion

We performed RNA-Seq using whole blood of patients with ARDS after HSCT and compared the transcriptomic profile to that of non-HSCT ARDS patients. Our analysis identified a set of differentially expressed genes with the strongest differences in pathways related to innate immune responses to pathogens and type I interferon signaling, all of which were upregulated in ARDS. Our findings raise the question of whether there may be an immune dysregulation post HSCT that predisposes subjects to lung injury or ARDS. Recently, Nick et al. found that high- and low- blood expression of a set of interferon-stimulated genes (*MX1*, *IFIT1*, and *ISG15*) identified a set of ARDS patients with worse outcomes. Morrell et al., analyzed peripheral blood monocyte gene expression by microarray and found increased interferon signaling in ARDS patients with a favorable outcome (i.e. more ventilator-free days) compared to ARDS patients with fewer ventilator-free days [34]. Thus, it is enticing to hypothesize that modulation of interferon signaling pathways represents a treatment approach for HSCT subjects, but additional data are required to support such a strategy. Interestingly, a recently completed phase 3 trial of human interferon beta 1a for moderate-to-severe ARDS did not find a significant difference in the primary composite endpoint of mortality and ventilator free days [35, 36]. Although the administration of interferon beta 1a was not efficacious in this population of unselected patients, it is possible that assessing whether interferon signaling pathways are activated in ARDS patients might provide a more targeted approach to treatment.

Our study is the first to use RNA-Seq in order to determine whether the specific ARDS subphenotype of HSCT-ARDS has a unique peripheral blood gene expression profile. Our patients were carefully selected from a single center and although sample size was small, this proof-of-concept study provides insights that are valuable to design future studies. We chose age- and sex-matched subjects to limit heterogeneity for this initial study, though we note that this may limit the generalizability of our findings. Another limitation of our study is the use of peripheral blood RNA, which is derived from a mixture of cell types. Although there were no significant differences in differential cell counts between groups, it is possible that gene expression profiles could be affected by subtle changes in cell maturity between groups. For example, blood gene expression profiles of patients with ARDS may have been dominated by systemic inflammation when compared to subjects without ARDS [37]. We focused our study on two subgroups of ARDS patients, presumably with similar degrees of inflammation, in order to identify differences in gene expression that may explain heterogeneity amongst different types of ARDS patients. Our two groups were similar in demographic characteristics and severity of lung injury and overall illness, but still demonstrated clinical heterogeneity. While our results suggest that gene expression differences were not due to HSCT itself, we are unable to rule out the effect of potential confounders such as immunosuppressant medications. Notably, all of the patients in the ARDS-HSCT group were being treated with moderate-to-high dose corticosteroids at the time of inclusion, whereas half of the non-HSCT ARDS patients were on moderate dose corticosteroids. Another potential confounder is the possibility of undiagnosed infections. To address this issue, we analyzed reads from our RNA-Seq data that did not map to the human genome and performed taxonomic classification to look for the presence of common pathogens. Although our data did not suggest the presence of an undiagnosed bloodstream infection, this analysis was limited by the sample size, the fact that some respiratory pathogens might not exhibit a bloodstream signature, and the fact that the majority of bacterial RNA are not polyadenylated and may have been eliminated during the library preparation process. Finally, our samples were collected over the course of five years during which a new definition of ARDS was adopted, [1] and thus, it is possible that multiple definitions could have unintentionally impacted our results.

Patients who have undergone HSCT are at heightened risk for a number of types of lung injury including pulmonary infections as well as non-infectious lung injury such as drug-induced pneumonitis, diffuse alveolar hemorrhage, and idiopathic pneumonia syndrome. Given the lack of well-defined diagnostic criteria for many of these entities, other investigators have focused on the epidemiology of the most severe form of lung injury (i.e. ARDS) regardless of the etiology and found that ARDS after allo-HSCT is common and has high mortality [12]. We used a similar strategy for our study choosing not to focus on the proximal cause of lung injury. One limitation of this strategy is that it is difficult to determine whether the transcriptomic changes in the ARDS-HSCT population reflect the different underlying etiologies of ARDS, differential host responses, or most likely both. Interestingly, the majority of patients in the HSCT-ARDS group were suspected of having idiopathic pneumonia syndrome and received treatment with etanercept. While the effect of etanercept on peripheral blood gene expression in critical illness is not known, studies in autoimmune diseases have found differential regulation of interferon pathways. For example, *OAS1*, *OAS2*, *OAS3*, and *IFI44L* appear to be down-regulated by etanercept in responders to treatment for psoriasis [38]. However, in a set of patients treated with infliximab for rheumatoid arthritis, treatment induced an increase in interferon response genes including *OAS1* and *OAS2* in a subset of patients who had poor clinical response; [39] a subsequent study including patients on a variety of anti-TNF therapies including etanercept also found divergent responses in interferon profiles, though did not find an association with clinical response [40].

## Conclusions

ARDS is a heterogeneous syndrome, with a diverse set of risk factors, etiologies, and outcomes. Our study is the first to identify a potential difference in gene expression that distinguishes ARDS-HSCT from ARDS, representing changes in immune response and interferon signaling pathways. Our data, along with existing literature, suggest that there is a spectrum of dysregulated interferon signaling underlying lung injury and further supports the notion that improved phenotyping of ARDS subgroups is critical for designing targeted treatments for this devastating syndrome. Importantly, our data raises the intriguing question of whether there exists a modifiable immune deficiency post-HSCT that ameliorates lung injury. Future studies to examine these areas are critically important.

## Additional files

Additional file 1: **Table S1.** Individual gene names from David functional clustering analysis. **Table S2.** Top results from DAVID Functional Annotation Clustering, excluding 2 subjects with diagnosed influenza **Table S3.** Number of reads per sample assignable to a virus. (DOCX 24 kb)

Additional file 2: **Figure S1.** Transcript levels of several of the most differentially expressed genes were measured by qPCR to confirm changes found by RNA-seq. *n* = 3/group, *p < 0.05 vs ARDS by unpaired t-test. **Figure S2.** Taxonomic classification of unmapped reads within each patient sample after human read removal. Relative read count indicates the fraction of reads belonging to a given genus out of all reads classified to at least genus-level specificity. Genera with minimum read fraction of 2% within a sample are displayed (otherwise collapsed into “Other”). No dramatic classwise differences are observed, and the lack of a recurrent abundant pathogen unique to ARDS-SCT patients suggests that undiagnosed bloodstream infection is an unlikely cause of ARDS in these SCT patients. (DOCX 549 kb)

## Abbreviations

allo HSCT: Allogeneic hematopoeitic stem cell transplant
ARDS: Acute respiratory distress syndrome
ICU: Intensive care unit
RNA-seq: RNA sequencing
