# Supplementary material for: Whole blood RNA sequencing reveals a unique transcriptomic profile in patients with ARDS following hematopoietic stem cell transplantation
Source: Respir Res. 2019 Jan 21;20:15. doi: 10.1186/s12931-019-0981-6 (PMC6341764; doi:10.1186/s12931-019-0981-6)
Supplement: Supplementary file 1 — Table S1. Individual gene names from David functional clustering analysis. Table S2. Top results from DAVID Functional Annotation Clustering, excluding 2 subjects with diagnosed influenza Table S3. Number of reads per sample assignable to a virus. (DOCX 24 kb) [file 12931_2019_981_MOESM1_ESM.docx]

**Supplementary materials**

**Additional file 1: Table S1. Individual gene names from David functional clustering analysis.**

| GO: Antiviral Defense |  |
| --- | --- |
| ID | **Gene Name** |
| ENSG00000089127 | 2'-5'-oligoadenylate synthetase 1(OAS1) |
| ENSG00000111335 | 2'-5'-oligoadenylate synthetase 2(OAS2) |
| ENSG00000111331 | 2'-5'-oligoadenylate synthetase 3(OAS3) |
| ENSG00000135114 | 2'-5'-oligoadenylate synthetase like(OASL) |
| ENSG00000107201 | DExD/H-box helicase 58(DDX58) |
| ENSG00000137628 | DExD/H-box helicase 60(DDX60) |
| ENSG00000108771 | DExH-box helicase 58(DHX58) |
| ENSG00000138646 | HECT and RLD domain containing E3 ubiquitin protein ligase 5(HERC5) |
| ENSG00000187608 | ISG15 ubiquitin-like modifier(ISG15) |
| ENSG00000157601 | MX dynamin like GTPase 1(MX1) |
| ENSG00000101347 | SAM and HD domain containing deoxynucleoside triphosphate triphosphohydrolase 1(SAMHD1) |
| ENSG00000197381 | adenosine deaminase, RNA specific B1(ADARB1) |
| ENSG00000243811 | apolipoprotein B mRNA editing enzyme catalytic subunit 3D(APOBEC3D) |
| ENSG00000128394 | apolipoprotein B mRNA editing enzyme catalytic subunit 3F(APOBEC3F) |
| ENSG00000130303 | bone marrow stromal cell antigen 2(BST2) |
| ENSG00000130813 | chromosome 19 open reading frame 66(C19orf66) |
| ENSG00000055332 | eukaryotic translation initiation factor 2 alpha kinase 2(EIF2AK2) |
| ENSG00000117228 | guanylate binding protein 1(GBP1) |
| ENSG00000117226 | guanylate binding protein 3(GBP3) |
| ENSG00000137959 | interferon induced protein 44 like(IFI44L) |
| ENSG00000185745 | interferon induced protein with tetratricopeptide repeats 1(IFIT1) |
| ENSG00000119922 | interferon induced protein with tetratricopeptide repeats 2(IFIT2) |
| ENSG00000119917 | interferon induced protein with tetratricopeptide repeats 3(IFIT3) |
| ENSG00000152778 | interferon induced protein with tetratricopeptide repeats 5(IFIT5) |
| ENSG00000115267 | interferon induced with helicase C domain 1(IFIH1) |
| ENSG00000185507 | interferon regulatory factor 7(IRF7) |
| ENSG00000110944 | interleukin 23 subunit alpha(IL23A) |
| ENSG00000140464 | promyelocytic leukemia(PML) |
| ENSG00000115415 | signal transducer and activator of transcription 1(STAT1) |
| ENSG00000170581 | signal transducer and activator of transcription 2(STAT2) |
|  |  |
| GO: Response to virus |  |
| ID | **Gene Name** |
| ENSG00000089127 | 2'-5'-oligoadenylate synthetase 1(OAS1) |
| ENSG00000111335 | 2'-5'-oligoadenylate synthetase 2(OAS2) |
| ENSG00000111331 | 2'-5'-oligoadenylate synthetase 3(OAS3) |
| ENSG00000135114 | 2'-5'-oligoadenylate synthetase like(OASL) |
| ENSG00000107201 | DExD/H-box helicase 58(DDX58) |
| ENSG00000137628 | DExD/H-box helicase 60(DDX60) |
| ENSG00000108771 | DExH-box helicase 58(DHX58) |
| ENSG00000157601 | MX dynamin like GTPase 1(MX1) |
| ENSG00000130303 | bone marrow stromal cell antigen 2(BST2) |
| ENSG00000055332 | eukaryotic translation initiation factor 2 alpha kinase 2(EIF2AK2) |
| ENSG00000137965 | interferon induced protein 44(IFI44) |
| ENSG00000185745 | interferon induced protein with tetratricopeptide repeats 1(IFIT1) |
| ENSG00000119922 | interferon induced protein with tetratricopeptide repeats 2(IFIT2) |
| ENSG00000119917 | interferon induced protein with tetratricopeptide repeats 3(IFIT3) |
| ENSG00000115267 | interferon induced with helicase C domain 1(IFIH1) |
| ENSG00000185507 | interferon regulatory factor 7(IRF7) |
| ENSG00000143324 | xenotropic and polytropic retrovirus receptor 1(XPR1) |
|  |  |
| GO: Heme biosynthesis |  |
| ID | Gene Name |
| ENSG00000158578 | 5'-aminolevulinate synthase 2(ALAS2) |
| ENSG00000181873 | IBA57 homolog, iron-sulfur cluster assembly(IBA57) |
| ENSG00000169877 | alpha hemoglobin stabilizing protein(AHSP) |
| ENSG00000090013 | biliverdin reductase B(BLVRB) |
| ENSG00000080819 | coproporphyrinogen oxidase(CPOX) |
| ENSG00000066926 | ferrochelatase(FECH) |
| ENSG00000256269 | hydroxymethylbilane synthase(HMBS) |
| ENSG00000143224 | protoporphyrinogen oxidase(PPOX) |
| ENSG00000013306 | solute carrier family 25 member 39(SLC25A39) |
|  |  |
| GO: Hereditary hemolytic anemia |  |
| ID | **Gene Name** |
| ENSG00000112077 | Rh-associated glycoprotein(RHAG) |
| ENSG00000188157 | agrin(AGRN) |
| ENSG00000029534 | ankyrin 1(ANK1) |
| ENSG00000172331 | bisphosphoglycerate mutase(BPGM) |
| ENSG00000108091 | coiled-coil domain containing 6(CCDC6) |
| ENSG00000158856 | dematin actin binding protein(DMTN) |
| ENSG00000159023 | erythrocyte membrane protein band 4.1(EPB41) |
| ENSG00000166947 | erythrocyte membrane protein band 4.2(EPB42) |
| ENSG00000001084 | glutamate-cysteine ligase catalytic subunit(GCLC) |
| ENSG00000117394 | solute carrier family 2 member 1(SLC2A1) |
| ENSG00000004939 | solute carrier family 4 member 1 (Diego blood group)(SLC4A1) |
| ENSG00000163554 | spectrin alpha, erythrocytic 1(SPTA1) |
|  |  |
| Herpes simplex infection |  |
| ID | **Gene Name** |
| ENSG00000089127 | 2'-5'-oligoadenylate synthetase 1(OAS1) |
| ENSG00000111335 | 2'-5'-oligoadenylate synthetase 2(OAS2) |
| ENSG00000111331 | 2'-5'-oligoadenylate synthetase 3(OAS3) |
| ENSG00000135114 | 2'-5'-oligoadenylate synthetase like(OASL) |
| ENSG00000169245 | C-X-C motif chemokine ligand 10(CXCL10) |
| ENSG00000107201 | DExD/H-box helicase 58(DDX58) |
| ENSG00000157601 | MX dynamin like GTPase 1(MX1) |
| ENSG00000099804 | cell division cycle 34(CDC34) |
| ENSG00000086232 | eukaryotic translation initiation factor 2 alpha kinase 1(EIF2AK1) |
| ENSG00000055332 | eukaryotic translation initiation factor 2 alpha kinase 2(EIF2AK2) |
| ENSG00000117228 | guanylate binding protein 1(GBP1) |
| ENSG00000185745 | interferon induced protein with tetratricopeptide repeats 1(IFIT1) |
| ENSG00000204010 | interferon induced protein with tetratricopeptide repeats 1B(IFIT1B) |
| ENSG00000115267 | interferon induced with helicase C domain 1(IFIH1) |
| ENSG00000185507 | interferon regulatory factor 7(IRF7) |
| ENSG00000114030 | karyopherin subunit alpha 1(KPNA1) |
| ENSG00000140464 | promyelocytic leukemia(PML) |
| ENSG00000213639 | protein phosphatase 1 catalytic subunit beta(PPP1CB) |
| ENSG00000115415 | signal transducer and activator of transcription 1(STAT1) |
| ENSG00000170581 | signal transducer and activator of transcription 2(STAT2) |
| ENSG00000187555 | ubiquitin specific peptidase 7(USP7) |

**Additional file 1: Table S2. Top results from DAVID Functional Annotation Clustering, excluding 2 subjects with diagnosed influenza**

| Cluster | Enrichment score | Example pathway | Count | Adjusted P-value |
| --- | --- | --- | --- | --- |
| 1 | 9.25 | Uniprot: Antiviral defense | 24 | 3.4x10-20 |
| 2 | 3.69 | GO: double-stranded RNA binding | 11 | 7.3x10-7 |
| 3 | 2.41 | GO: negative regulation of type I interferon production | 6 | 5.4x10-3 |
| 4 | 1.84 | KEGG: RIG-I like receptor signaling pathway | 6 | 4.6x10-2 |

**Additional file 1: Table S3. Number of reads per sample assignable to a virus.**

| Sample | Category | Viral reads |
| --- | --- | --- |
| A | ARDS | 97 reads aligning to EBV |
| B | ARDS | 9 reads assignable to EBV |
| C | ARDS-HSCT | 4 reads assignable to a torquetenovirus |
| D | Sepsis | 9 reads assignable to a torquetenovirus |
| All others | All | 0 reads assignable to a virus |
