# Supplementary material for: Whole blood RNA sequencing reveals a unique transcriptomic profile in patients with ARDS following hematopoietic stem cell transplantation
Source: Respir Res. 2019 Jan 21;20:15. doi: 10.1186/s12931-019-0981-6 (PMC6341764; doi:10.1186/s12931-019-0981-6)
Supplement: Supplementary file 2 — Figure S1. Transcript levels of several of the most differentially expressed genes were measured by qPCR to confirm changes found by RNA-seq. n = 3/group, *p < 0.05 vs ARDS by unpaired t-test. Figure S2. Taxonomic classification of unmapped reads within each patient sample after human read removal. Relative read count indicates the fraction of reads belonging to a given genus out of all reads classified to at least genus-level specificity. Genera with minimum read fraction of 2% within a sample are displayed (otherwise collapsed into “Other”). No dramatic classwise differences are observed, and the lack of a recurrent abundant pathogen unique to ARDS-SCT patients suggests that undiagnosed bloodstream infection is an unlikely cause of ARDS in these SCT patients. (DOCX 549 kb) [file 12931_2019_981_MOESM2_ESM.docx]

**A**

**B**

**C**

**D**

**Figure 3**. Transcript levels of several of the most differentially expressed genes were measured by qPCR to confirm changes found by RNA-seq. n=3/group, *p<0.05 vs ARDS by unpaired T-test.

**Additional file 2: Figure S1**. Transcript levels of several of the most differentially expressed genes were measured by qPCR to confirm changes found by RNA-seq. n=3/group, *p<0.05 vs ARDS by unpaired t-test.

**
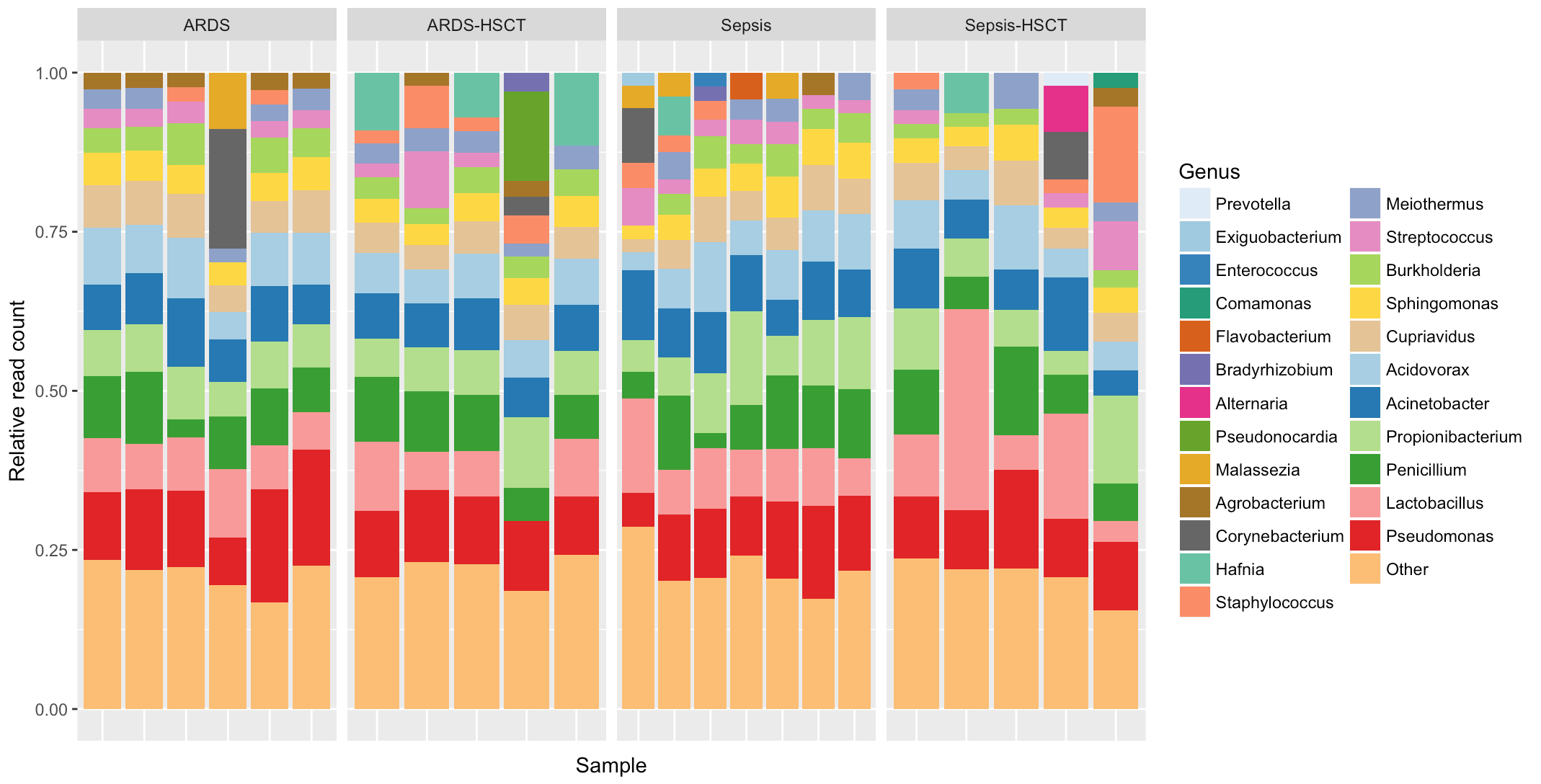
**

**Additional file 2: Figure S2**. Taxonomic classification of unmapped reads within each patient sample after human read removal. Relative read count indicates the fraction of reads belonging to a given genus out of all reads classified to at least genus-level specificity. Genera with minimum read fraction of 2% within a sample are displayed (otherwise collapsed into “Other”). No dramatic classwise differences are observed, and the lack of a recurrent abundant pathogen unique to ARDS-SCT patients suggests that undiagnosed bloodstream infection is an unlikely cause of ARDS in these SCT patients.
